# Laser Direct Writing of Setaria Virids-Inspired Hierarchical Surface with TiO_2_ Coating for Anti-Sticking of Soft Tissue

**DOI:** 10.3390/mi15091155

**Published:** 2024-09-15

**Authors:** Qingxu Zhang, Yanyan Yang, Shijie Huo, Shucheng Duan, Tianao Han, Guang Liu, Kaiteng Zhang, Dengke Chen, Guang Yang, Huawei Chen

**Affiliations:** 1 School of Mechanical Engineering, Hebei University of Science and Technology, Shijiazhuang 050018, China; 15631817192@163.com (Q.Z.); 17367837787@163.com (S.H.); 15174890829@163.com (S.D.); hf0402101211@163.com (T.H.); y_guang@126.com (G.Y.); 2 960 Hospital of the PLA, Tai’an 271000, China; yang1665319@163.com; 3 College of Transportation, Ludong University, Yantai 264025, China; zkteng90@163.com (K.Z.); 716316hay@163.com (D.C.); 4 School of Mechanical Engineering and Automation, Beihang University, Beijing 100191, China

**Keywords:** setaria virids, laser direct writing, hierarchical micro texturing, TiO_2_ coatings, anti-sticking, soft tissue

## Abstract

In minimally invasive surgery, the tendency for human tissue to adhere to the electrosurgical scalpel can complicate procedures and elevate the risk of medical accidents. Consequently, the development of an electrosurgical scalpel with an anti-sticking coating is critically important. Drawing inspiration from nature, we identified that the leaves of Setaria Virids exhibit exceptional non-stick properties. Utilizing this natural surface texture as a model, we designed and fabricated a specialized anti-sticking surface for electrosurgical scalpels. Employing nanosecond laser direct writing ablation technology, we created a micro-nano textured surface on the high-frequency electrosurgical scalpel that mimics the structure found on Setaria Virids leaves. Subsequently, a TiO_2_ coating was deposited onto the ablated scalpel surface via magnetron sputtering, followed by plasma-induced hydrophobic modification and treatment with octadecyltrichlorosilane (OTS) to enhance the surface’s affinity for silicone oil, thereby constructing a self-lubricating and anti-sticking surface. The spreading behavior of deionized water, absolute ethanol, and dimethyl silicone oil on this textured surface is investigated to confirm the effectiveness of the self-lubrication mechanism. Furthermore, the sticking force and quality are compared between the anti-sticking electrosurgical scalpel and a standard high-frequency electrosurgical scalpel to demonstrate the efficacy of the nanosecond laser-ablated micro-nano texture in preventing sticking. The findings indicate that the self-lubricating anti-sticking surface fabricated using this texture exhibits superior anti-sticking properties.

## 1. Introduction

High-frequency electrosurgical scalpels have emerged as indispensable instruments in contemporary operating theaters, thanks to their superior performance. They are extensively utilized across various surgical disciplines, including general, thoracic and cardiovascular, neuro, urological, and plastic surgeries [1,2,3,4,5]. The deployment of high-frequency electrosurgical scalpels significantly reduces surgical duration and effectively decreases the incidence of post-operative complications [6,7,8], thereby enhancing overall surgical outcomes and expediting patient recovery. However, their use is not without challenges. The scalpels’ operation relies on tissue cutting through heat generation by high-frequency current, leading to an extremely high temperature. Consequently, tissue sticking, which can result in enlarged wounds and bleeding, remains an issue, posing a threat to patient safety [9]. To mitigate this issue, developing a functionalized surface with exceptional anti-sticking properties on the electrosurgical scalpel is a key strategy.

Efforts to enhance the anti-sticking attributes of electrosurgical scalpels encompass physical and chemical approaches. Physical methods predominantly involve the use of nanosecond/picosecond lasers to fabricate micro-nano textures on the scalpel’s surface [10,11,12,13,14,15,16,17,18,19,20,21]. For instance, Zhang Li and her team employed laser texturing technology to create bionic micro-nano composite structures on the surface of Co28Cr6Mo alloy. Furthermore, they successfully developed a functionalized surface with outstanding corrosion resistance and superlubricity characteristics by utilizing a modification process involving polydimethylsiloxane solution and silicone oil [22]. Following this, Zhou Haonan and colleagues ingeniously designed an anti-icing slippery liquid-infused porous surface (SLIPSs), drawing inspiration from the sleek inner surface of pitcher plants. Utilizing a dip-coating technique, they successfully created a hydrophobic silica layer that is grafted with long-chain organic molecules. Following the infusion of an oil phase, a three-tiered structure emerges, mirroring the internal architecture of pitcher plants, and this innovative surface demonstrates exceptional capabilities in resisting ice formation [23]. Chemical methods include the application of polymer coatings and sol-gel processes [24,25,26,27,28], such as PET [29], fluorinated polymers [30], silicones [31,32,33,34], and cyclosilazanes [35,36] with low surface energy. Research indicates that both approaches have limitations. Micro-nano textures produced by nanosecond/picosecond laser technology, while providing some level of anti-sticking, lack durability and are prone to wear during scalpel use, leading to the loss of anti-sticking capabilities. Additionally, traditional chemical methods for anti-sticking coatings may offer initial protection but are unreliable, particularly in high-temperature conditions where coatings may degrade and release toxic substances. This not only endangers patients but also risks the health of medical professionals. Therefore, there is an urgent need to investigate a novel method that can effectively address the anti-sticking challenges associated with electrosurgical scalpels [37,38].

**Figure 1:**
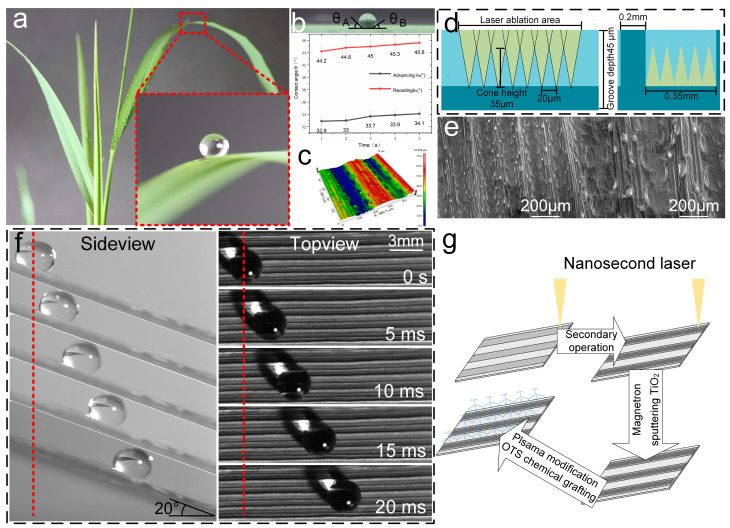
(**a**) The morphology of Setaria Virids and the sticking state of dewdrops on the surface of Setaria Virids leaves. (**b**) The front contact angle θ_A_ and the rear contact angle θ_B_ of a droplet on the surface of a horizontally placed Setaria Virids leaf. (**c**) White light interference morphology characterization of micro-nano textures processed by nanosecond laser. (**d**) Ideal illustration of micro-nano textures prepared using nanosecond laser. (**e**) Scanning Electron Microscopy (SEM) characterization of the microstructure on the surface of Setaria Virids leaves. (**f**) The flow of a droplet on the surface of a Setaria Virids leaf placed at an inclination of 20°. (**g**) A mechanism illustration showing the preparation of a functionalized surface with anti-sticking properties by creating a microgroove–micropillar composite texture using a nanosecond laser, followed by Plasma modification and a self-assembled molecular layer coating.

Emulating nature, or learning from the natural world, is a pivotal strategy in scientific innovation. It involves a meticulous examination and study of the myriad phenomena and principles found in nature to harness inspiration and apply it to our design endeavors, with the goal of achieving more optimal results. Through careful observation of the leaves of Setaria Virids, we have identified a distinctive microgroove–columnar texture on their surfaces (Figure 1c,e). This texture enables liquid droplets to roll effortlessly across the leaf surface without lingering, substantially reducing the buildup of soil and stains [39]. This attribute not only maintains the cleanliness of the Setaria Virids leaves but also ensures their capacity for photosynthesis, thereby sustaining the energy required for growth.

In our quest to develop new methods for combating sticking in electrosurgical scalpels, we have drawn inspiration from the micro-nano textures of the natural world. We are exploring the application of these textures to the surface of electrosurgical scalpels using nanosecond laser technology. Subsequently, magnetron sputtering was employed to uniformly deposit TiO_2_ onto the functionalized surface, forming a TiO_2_ thin film, combined with the infusion of silicone oil to achieve anti-sticking and antimicrobial properties. TiO_2_ thin films have emerged as a highly promising and valuable asset in the realm of disinfection and sterilization. This innovative material has captured extensive interest owing to its exceptional photocatalytic sterilization properties. When exposed to ultraviolet light, TiO_2_ thin films are capable of generating a substantial number of electrons and holes, which trigger a cascade of photocatalytic reactions that effectively deactivate bacteria, viruses, and a range of other pathogenic micro-organisms. The distinctive photo-catalytic attributes of TiO_2_ thin films pave the way for extensive applications in the food processing and disinfection industries. Especially within the healthcare sector, the utilization of TiO_2_ thin films offers an efficient method for sterilizing medical instruments and hospital settings, markedly diminishing the incidence of hospital-acquired infections and ensuring a more secure environment for both patients and healthcare personnel. We have engineered a uniform array of microgroove textures, with five rows of microcolumns uniformly spaced at the base of each groove, designed to facilitate the directional and spontaneous diffusion of the lubricant along the microgrooves. This creates a lubricating film when the scalpel interacts with tissue, thereby reducing sticking [40,41]. Dimethyl silicone oil was chosen as the lubricant due to its ability to spread easily at high temperatures, its biologically inert nature, and its excellent hydrophobic properties [42,43,44]. To enhance the sticking of dimethyl silicone oil to the micro-nano textured surface, we employed a chemical technique, grafting an OTS (octadecyltrichlorosilane) self-assembled coating onto the self-lubricating anti-sticking surface. This forms a nanometers-thick siloxane bond, which not only improves the stability of the lubricant at high temperatures but also increases its longevity on the scalpel surface [45].

To validate the anti-sticking efficacy of the functionalized surface with micro-nano textures, we conducted tests on the unidirectional flow and reverse gravity spreading of liquids, and employed a push–pull force gauge to measure sticking force and sticking quantity. These experiments are aimed at thoroughly assessing the anti-sticking capabilities of the developed self-lubricating anti-sticking surfaces, providing a scientific foundation for further refinement and application. Through these extensive research efforts and testing, we aspire to deliver an efficient and dependable anti-sticking solution for electrosurgical scalpels, thereby enhancing the safety and efficiency of surgical procedures.

## 2. Materials and Methods

### 2.1. Materials

The experimental apparatus utilized in this study comprises a nanosecond laser-marking machine (Beijing Leijieming Laser Technology Development Company Limited. (Beijing, China)), a microscope, a Canon 90D camera (Canon Inc., Ota City, Japan), a high-speed camera (Hefei Zhongke Junda Vision Technology Co., Ltd. (Hefei, China)), a water bath heating platform, a Plasma ion cleaner (PlasmaTechnology GmbH, Herrenberg, Germany), a magnetron sputtering apparatus, a precision electronic balance (with a precision of 0.001 g), an in-house-built platform for measuring pushing and pulling forces, related equipment for high-frequency electrosurgical scalpels (Beijing Yingjiahua Technology Company Limited. (Beijing, China)), and a nitrogen gas generator. The materials include fresh, complete Setaria Virids leaves, 316 L stainless steel electrosurgical scalpel blades (with dimensions of 50 mm × 2.6 mm × 0.8 mm), and 316 L stainless steel sheets (with dimensions of 50 mm × 50 mm × 1 mm), The experimental substrates were provided by Beijing ENJOY Technologies Company Limited (Beijing, China). The chemicals involved are HCl (hydrochloric acid), H_2_SO_4_ (concentrated sulfuric acid), OTS (octadecyltrichlorosilane), dimethyl silicone oil with a viscosity of 10 cs, nitrogen gas, deionized water, anhydrous ethanol, fluorescein sodium, and Rhodamine B. All the chemical reagents used in the experiment were supplied by Beijing Chemical Works (Beijing, China). Fresh, homogeneous in vitro pig liver was selected as the soft tissue material for testing anti-sticking force and quantity.

The Setaria Virids leaves were cut into rectangular blocks measuring 0.7 cm × 0.5 cm and placed under a scanning electron microscope (SEM) for imaging at magnifications of 150× and 500× to capture the SEM images of the surface’s microgroove–columnar texture (Figure 1e). The observation revealed that the surface of the Setaria Virids leaves exhibited a microgroove–columnar composite texture, with microcolumns evenly distributed on the convex platforms. It is speculated that this texture is designed to optimize the liquid flow properties of the leaf surface, such as reducing the sticking force of water droplets and promoting rainwater runoff, thus minimizing the accumulation of soil stains on the leaves and ensuring sufficient photosynthesis.

### 2.2. Fabrication of Electrodes with Hierachical Micro-Nano Array Structure

Utilizing a nanosecond laser direct-write ablation technique, a uniform microgroove-microcolumn composite texture was fabricated on the surface of an electrosurgical scalpel blade crafted from 316 L stainless steel, with microcolumns arrayed evenly at the base of each groove.

**Figure 2:**
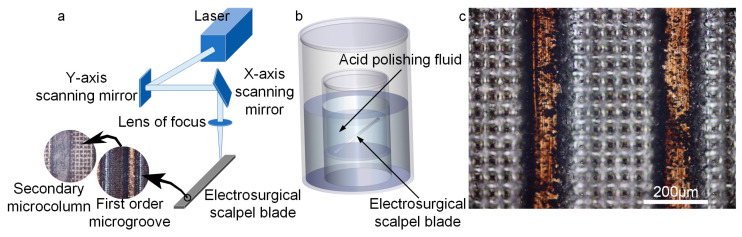
(**a**) Mechanism diagram of laser processing surface micro-nano texture. (**b**) Removal of sharp tips by water bath heating. (**c**) Microscopic observation of the processed surface micro-nano texture.

The process began by fully submerging the scalpel in anhydrous ethanol and subjecting it to an ultrasonic cleaning cycle at room temperature for three minutes to eliminate surface impurities. The scalpel was then sequentially rinsed with anhydrous ethanol and deionized water, followed by thorough drying under a stream of moving air. After cleaning, the scalpel was positioned at various angles (0°, 5°, 15°, 30°) relative to the horizontal plane on the laser processing table (Figure 2a).

The laser marking machine was initiated with the following settings: a laser power of 40%, a frequency of 40 KHz, a speed of 1000 mm/s, a line spacing of 0.02 mm, and a cycle count of 40. These parameters were used to create the primary microgroove texture. The sample, after its initial processing, was immersed in a solution composed of concentrated sulfuric acid, concentrated hydrochloric acid, and water in a ratio of 7:2:1, and then subjected to a water bath at 60 °C for 20 min. This step was crucial for the removal of surface irregularities and any molten residue resulting from the laser ablation.

Following the etching process, the sample was returned to the same position on the laser processing table. A secondary array of uniformly distributed microcolumns was then ablated using refined laser parameters: a power of 72%, a frequency of 50 KHz, a speed of 2500 mm/s, a line spacing of 0.05 mm, and a cycle count of 12, resulting in the desired composite texture (Figure 2c).

**Figure 3:**
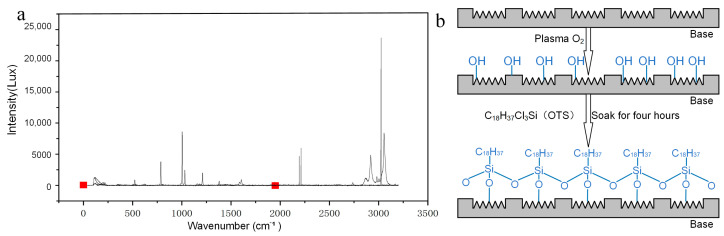
(**a**) Successful OTS chemical grafting was confirmed by Raman spectroscopy. (**b**) Chemical mechanism diagram of OTS self-assembly.

Utilizing magnetron sputtering technology, a magnetron ion sputtering system was employed to conduct ion sputtering treatment on the electrosurgical scalpel, which features a functionalized surface. Before the sputtering process, the scalpel was meticulously cleaned in an ultrasonic cleaning machine to ensure the surface was free of contaminants that could compromise the adhesion of TiO_2_ to the functionalized surface. This step was crucial for the formation of a uniform and continuous TiO_2_ thin film. Experimental studies revealed that the optimal sputtering parameters were as follows: an oblique target sputtering configuration with a power setting of 90 w, a rotational speed of 20 rpm/min, and a coating duration of 50 min, resulting in a copper film thickness of roughly 50 nm for the most desirable outcomes. The TiO_2_-coated sample was then introduced into a Plasma cleaner, where it underwent a 3-min modification process under an environment characterized by 60 W RF power and a vacuum pressure of 60 Pa (Figure 3b). This modification step was employed to augment the surface’s content of hydroxyl groups (-OH), thereby enhancing the binding force of the micro-nano texture on the electrosurgical scalpel surface with OTS.

The Plasma-modified sample was then fully immersed in a toluene solution containing OTS at a concentration of 1 mmol/L for a duration of 4 h. Utilizing in situ Raman spectroscopy, this investigation elucidated the grafting mechanism of octadecyl trichlorosilane (OTS) molecules onto the functionalized substrate. The spectral data demonstrated a significant congruence in the vibrational peak intensities and wavenumbers between the OTS solution and the surface post-grafting, indicative of the formation of covalent silane bonds between OTS and the substrate interface. Furthermore, these observations corroborated the alterations in the surface morphology, which were consistent with the anticipated structural modifications. The spectroscopic analysis also confirmed the successful implementation of surface functionalities, such as the enhancement of hydrophobicity, and the reaction’s completeness and specificity were ascertained, with the absence of residual OTS monomers or side products, thereby furnishing direct molecular-level corroboration of the surface modification protocol (Figure 3a). Subsequently, any residual OTS on the surface was rinsed away with anhydrous toluene, and the sample was fully dried in an environment of nitrogen gas [38]. Finally, 5 μL of dimethyl silicone oil with a viscosity of 10cs was evenly dispensed onto the sample surface using a pipette, ensuring that the oil completely filled the voids within the micro-nano texture to establish a functionalized surface endowed with ultra-lubricant and anti-sticking properties.

### 2.3. Morphological Characterization

In the experimental phase, nanosecond laser ablation technology was utilized to create intricate micro-nano textures on the surface of an electrosurgical scalpel, endowing it with enhanced functionality. The primary microgrooves, etched by the nanosecond laser, exhibited precise dimensions of 350 μm in width and 50 μm in depth, engineered to facilitate the flow of lubricant.

Nested within each primary microgroove, an array of five secondary microcolumns was meticulously positioned. These microcolumns showcased a consistent height range of 30 to 40 μm and a base diameter varying between 45 and 65 μm. The strategic arrangement of these microcolumns was intended to improve the retention and directed spread of lubricant, thereby enhancing the scalpel’s performance. Moreover, the inter-groove ridges were designed to safeguard the integrity of the microcolumns during the scalpel’s operation, preventing damage that could arise from extended use and potentially compromising the non-stick properties.

**Figure 4:**
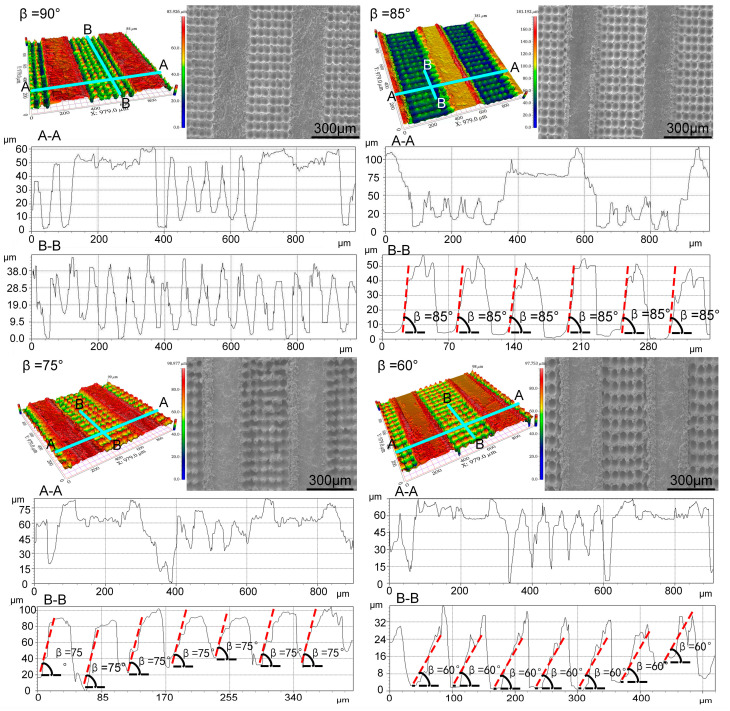
The white light interference morphology, longitudinal and transverse profile characterization, as well as SEM images of micro/nano texture were obtained at inclination angles β of 90°, 85°, 75°, and 60° between the oblique column and the horizontal plane. “A-A” and “B-B” denote the cross-sectional illustrations for the respective micro-nano textures, with “A-A” showcasing the horizontal profile and “B-B” the vertical profile.

To meticulously assess the formed micro-nano textures, state-of-the-art characterization techniques were employed, including a white light interferometer and a scanning electron microscope (SEM). The white light interferometer revealed the textures’ three-dimensional morphology, while the SEM provided detailed surface imagery at high resolution. By integrating the capabilities of both instruments, a thorough understanding of the microtextures’ microscopic architecture and geometric attributes was achieved. These characterization methods ensured the fabricated textures met the stringent requirements for non-stick functionality on electrosurgical scalpel surfaces, and they provided critical data for future performance and refinement efforts (Figure 4).

### 2.4. Unidirectional Transport Properties and Antigravity Flow Properties

In the experimental setup, dimethyl silicone oil with a viscosity of 10cs was selected as the lubricant of choice and was infused into the intricate voids of the micro-nano textures. The objective of this procedure was to harness the specialized design of the micro-nano textures to facilitate the effective retention and distribution of the lubricant across the surface of the electrosurgical scalpel. The dimethyl silicone oil was chosen for its outstanding stability at elevated temperatures, biocompatibility, and hydrophobic nature, which make it an ideal lubricating agent.

**Figure 5:**
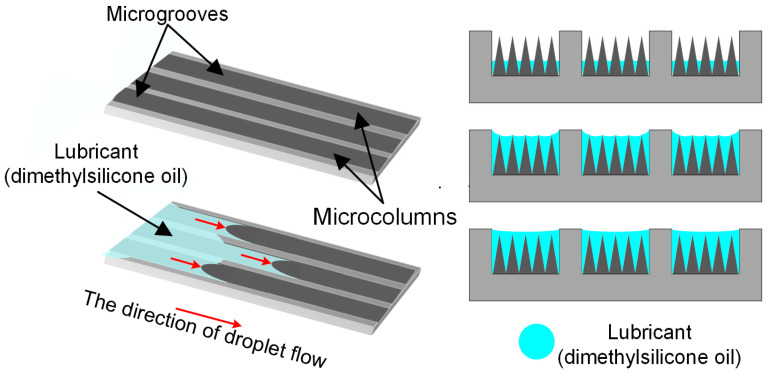
Self-lubricating anti-stick surface with added lubricant.

To ensure that the lubricant remained continuously and effectively engaged within the micro-nano textures, a novel reverse gravity spreading mechanism was developed. This mechanism enables the lubricant to autonomously refill the areas within the textures that are depleted. By leveraging the capillary action and surface tension inherent in the micro-nano structures, the lubricant is encouraged to flow spontaneously between the microgrooves and microcolumns. This self-regulating process ensures the formation of a persistent and even lubricating film during tissue contact, thereby minimizing sticking and friction, and enhancing the performance of the scalpel (Figure 5).

To investigate the reverse gravity spreading effect and unidirectional flow characteristics of functional surfaces enhanced with micro-nano textures under various liquids, we systematically examined these surfaces at a constant temperature of 25 °C. The experiment involved the use of deionized water, anhydrous ethanol, and dimethyl silicone oil as representative liquids. Drops of equal volume were introduced to the micro-nano textured surfaces to observe their reverse gravity spreading and unidirectional flow behaviors at different microcolumn angles. The goal was to elucidate how the geometric parameters of the micro-nano textures, the nature of the liquids, and the ambient conditions collaboratively affected the reverse gravity spreading and unidirectional flow dynamics. Deionized water and anhydrous ethanol were chosen for their widespread and typical use in biomedical contexts, while dimethyl silicone oil was considered due to its potential application as a lubricant.

The assessment of surface tension was based on the Young–Laplace pressure equation, in which Δ*p* denotes the pressure differential across the liquid interface, *R*_1_ and *R*_2_ are the radii of curvature of the liquid surface in planes orthogonal to the horizontal, and σ represents the surface tension of the liquid [46].

(1) $$∆p=\sigma (\frac{1}{R_{1}}+\frac{1}{R_{2}})$$

Young’s equation delineates the equilibrium condition at the interface between liquid, solid, and gaseous phases, where: γ represents the liquid’s surface tension, *θ* denotes the contact angle, $\gamma_{SG}$ characterizes the interfacial tension between the solid and the gas, and $\gamma_{SL}$ signifies the interfacial tension between the solid and the liquid.

(2) $$\gamma \cdot \cos\theta =\gamma_{SG}-\gamma_{SL}$$

On an ideal anti-sticking surface, the contact angle approaches 180 degrees, resulting in cos(*θ*) approaching 0. This implies that $\gamma_{SL}$ is nearly equal to $\gamma_{SG}$, indicating that the liquid almost does not wet the surface. The smaller the surface energy *E*, the poorer the hydrophilic properties of the surface. The formula for surface energy *E* is as follows:

(3) $$E=\gamma \cdot A_{drop}$$

Assuming the contact radius of the droplet on the blade surface is *r*, the surface area of the droplet (*A_drop_*) is calculated as follows:

(4) $$A_{drop}=\pi \cdot r^{2}$$

The calculated surface energy is below 10^−6^ J/m^2^, which suggests that the hydrophobic properties of the functionalized surface treated with dimethyl silicone oil are commendable.

**Figure 6:**
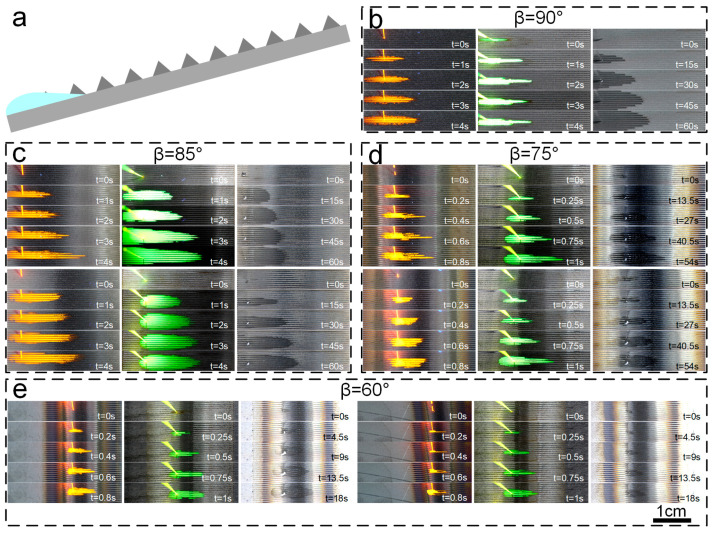
Antigravity spreading of deionized water (labeled with rhodamine B as orange yellow), anhydrous ethanol (labeled with fluorescein sodium as yellow green), and dimethylsilicone oil (colorless and transparent) on the texture of oblique columns with different inclination angles. (**a**) Antigravity spreading mechanism of liquid on micro/nano texture. (**b**) Antigravity spreading of liquid on a functional surface with a slant column angle of 90°. (**c**) Antigravity spreading of liquid on a functional surface with a slant column angle of 85°. (**d**) Antigravity spreading of liquid on a functional surface with a slant column angle of 75°. (**e**) Antigravity spreading of liquid on a functional surface with a slant column angle of 60°.

The observed reverse gravity spreading rates for the three liquids followed the sequence: *V*_(anhydrous ethanol)_ > *V*_(deionized water)_ > *V*_(dimethyl silicone oil)_. As the angle of inclination of the microcolumns was further reduced, the reverse gravity spreading velocities of all three liquids significantly increased (Figure 6). We also noticed that with an increase in the angle β between the slanted microcolumns and the horizontal plane, the unidirectional flow of the liquids became more distinct, always flowing in the direction of the column’s tilt. These findings not only provide critical experimental support for understanding how micro-nano textures regulate liquid flow but also offer essential guidance for refining the design of functional surfaces featuring such textures.

**Figure 7:**
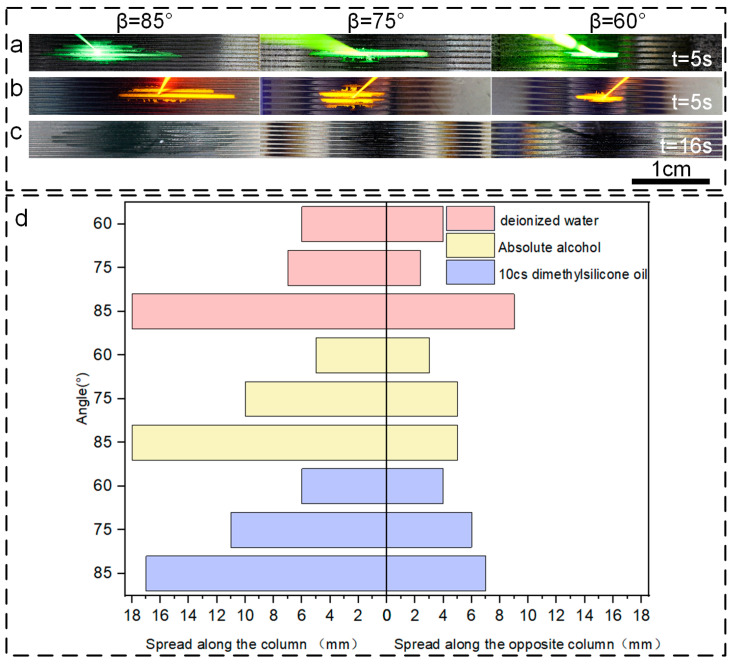
Unidirectional flow of the bulk on the texture of different slant column angles (90°, 85°, 75°, 60°). (**a**) Absolute ethanol (labeled with fluorescein sodium in yellow green). (**b**) Deionized water (labeled with rhodamine B in orange yellow). (**c**) 10cs dimethylsilicone oil (colorless and transparent). (**d**) Summary plot of one-way spreading data of the three liquids.

Our research revealed a strong correlation between the flow velocity of liquids and their surface tension σ. In particular, as the surface tension of a liquid rose, the corresponding radius of curvature diminished, resulting in a slower flow velocity. Since the surface tension order is *σ*_(anhydrous ethanol)_ < *σ*_(deionized water)_ < *σ*_(dimethyl silicone oil)_, the flow velocities followed the pattern *V*_(anhydrous ethanol)_ > *V*_(deionized water)_ > *V*_(dimethyl silicone oil)_. Moreover, variations in the angle β between the microcolumns and the horizontal plane had a pronounced impact on the liquids’ spreading velocities. As the microcolumn inclination decreased, the spreading velocities of all three liquids rose. Notably, on a 15° inclined surface, whether flowing with or against the column’s tilt, the order of reverse gravity spreading velocities was consistent with *V*_(anhydrous ethanol)_ > *V*_(deionized water)_ > *V*_(dimethyl silicone oil)_; and as the microcolumn inclination decreased further, the reverse gravity spreading velocities of all three liquids accelerated markedly (Figure 6). With an increase in the angle β, the unidirectional flow became increasingly evident, with all flows in the direction of the column’s tilt (Figure 7).

To delve into the intricacies of the reverse gravity spreading behavior of diverse liquid droplets on functionalized micro-nano textured surfaces with anti-sticking properties, a detailed schematic was developed using Visio software (2019) (Figure 6a). This illustration offers a clear visual representation of the droplet’s journey across the textured surface. Upon contact with the microgrooves at the surface, the droplets initially diffuse along the grooves’ bases. As they navigate through the array of microcolumns, the droplets are propelled against gravity by the increased Laplace pressure and the influence of additional capillary forces [46]. The droplets ascend along the direction of the microcolumn array. Moreover, the droplets experience a greater Laplace pressure in the direction of the microcolumn tilt compared to the opposite direction, resulting in faster spreading against gravity along the tilt of the microcolumns. This hypothesis is not only theoretically supported but is also borne out in practical applications (Figure 6). Experiments were conducted using Rhodamine B-labeled deionized water, fluorescein sodium-labeled anhydrous ethanol, and dimethyl silicone oil with a viscosity of 10cs to evaluate the reverse gravity spreading effects on the prepared functionalized surfaces. The experiment validated the calculated flow rates of different liquids (Figure 6b–e).

The examination of reverse gravity spreading has determined that an electrosurgical scalpel with a functionalized surface featuring micro-nano textures, produced via nanosecond laser technology, exhibits reverse gravity spreading properties when positioned at an angle α of 15° with respect to the horizontal plane. By comparing the reverse gravity spreading behavior of different liquids across micro-nano textures with varying inclined column angles β, it is evident that the smaller the β value, the more rapid the flow rate of the liquid. This corroborates the ability of the micro-nano textured functionalized surface, prepared using nanosecond laser technology, to facilitate the self-lubrication of the lubricant during surgical procedures when the electrosurgical scalpel is positioned at an angle less than 15° from the horizontal plane.

### 2.5. Cutting Experiment and Anti-Sticking Test

**Figure 8:**
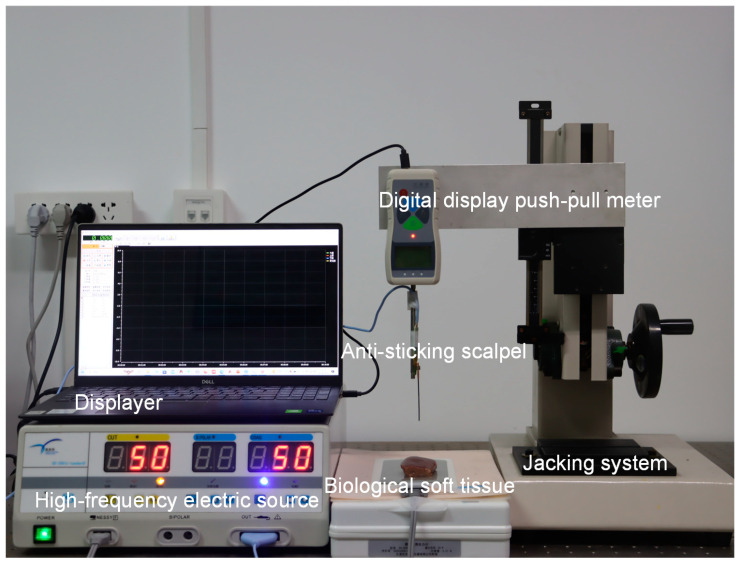
Sticking force measured by digital explicit push–pull dynamometer.

To assess the anti-sticking properties of the micro-nano textured functionalized surfaces, tissue cutting experiments were performed. Ensuring the precision and replicability of the results was paramount, hence stringent control over the experimental conditions was exercised to minimize any extraneous errors or influences. Fresh pork liver was initially trimmed into cubic samples measuring 2 cm on each side and then secured to an electrode plate positioned directly beneath the push–pull force gauge. The electrosurgical scalpel, equipped with the micro-nano textured functionalized surface, was mounted onto the measuring probe of the force gauge. To mimic actual surgical scenarios, the power supply for the high-frequency electrosurgical scalpel was adjusted to 50 W. The push–pull force gauge was utilized to determine the sticking force of the self-lubricating, anti-sticking surface (Figure 8).

In the course of the experiment, the handle of the push–pull force gauge was operated to control the electrosurgical scalpel, which was activated, as it made contact with the pork liver surface at a rate of 1 mm/s. The scalpel was then withdrawn at the same speed. The force registered by the gauge during the withdrawal phase constituted the sticking force. This approach facilitated a quantitative evaluation of the anti-sticking efficacy of the micro-nano textured functionalized surfaces.

During the application of the anti-sticking electrosurgical scalpel, a segment of the biological soft tissue in contact with it underwent vaporization, concurrently accompanied by the discharge of blood. Quantitative measurements reveal that: the scalpel width (*D*) is 2 mm, the scalpel travel speed (*v*) is 5 mm/s, the blood density (ρ) is 1050 kg/m^3^, the blood kinematic viscosity (*μ*) is 3.5 mPa·s.

Reynolds number formula:

(5) $$Re=\frac{\rho \cdot v\cdot D}{\mu }$$

Calculate the Reynolds number *Re* < 2000, is laminar flow, then the shear force calculation formula is:

(6) $$\tau =\frac{F}{A}=\frac{6\cdot \mu \cdot v}{D}$$

The formula for calculating the pressure *P* of the fluid on the scalpel surface is:

(7) $$P=\frac{1}{2}\rho v^{2}$$

By calculation, the shear force and pressure are below the safe value, which proves that the anti-stick electric scalpel with functional surface has good performance.

**Figure 9:**
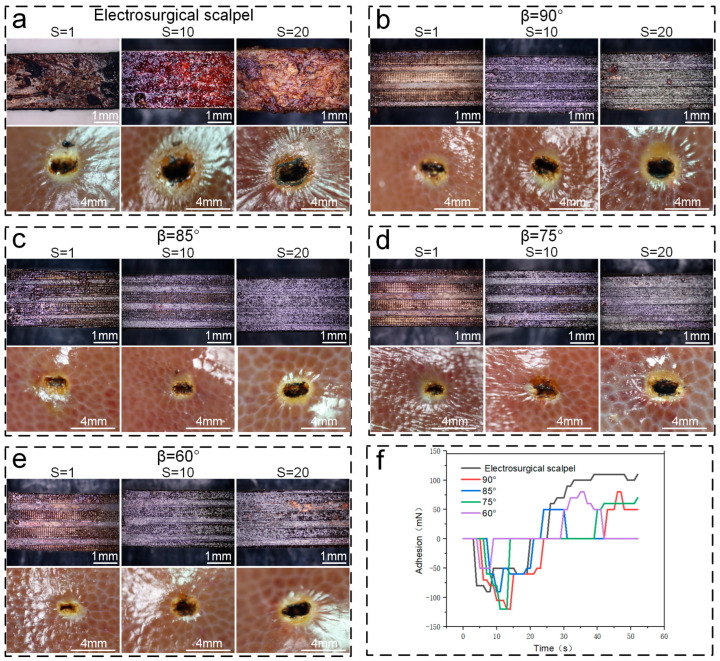
Sticking force and adhesion scale characteristics of high frequency electric scalpel and the inclination Angle β = 90°, 85°, 75°, and 60° between the upper oblique column of the electric scalpel and the horizontal plane. (**a**) When the operating power of the electric scalpel was 50 w, the amount of adhesion and thermal injury wound of fresh pig liver was cut with a high frequency electric scalpel (the number of electric cutting S = 1, 10, 20). (**b**) When the operating power of the electric scalpel was 50 w, the amount of adhesion and thermal injury wound of fresh pig liver was cut by the anti-stick electric scalpel with the angle of microcolumn β = 90° (the number of electric cutting S = 1, 10, 20). (**c**) When the electric scalpel working power was 50 w, the amount of adhesion and thermal injury wound of fresh pig liver was cut by the anti-stick electric scalpel with the angle of microcolumn β = 85° (the number of electric cutting S = 1, 10, 20). (**d**) When the operating power of electric scalpel was 50 w, the amount of adhesion and thermal injury wound of fresh pig liver was cut by the anti-stick electric scalpel with the angle β = 85°. The amount of adhesion and thermal injury wound of fresh pig liver was cut by the anti-stick electric scalpel with the angle of microcolumn β = 75° (the number of electric cutting S = 1, 10, 20). (**e**) When the operating power of the electric scalpel was 50 w, the amount of adhesion and thermal injury wound (electrotomy times S = 1, 10, 20) of fresh pig liver was cut with the anti-stick electrotome with micropillar angle β = 60°. (**f**) Comparison of adhesion forces between high frequency electrotome and different micropillar angle (90°, 85°, 75°, 60°).

Through meticulous measurements of sticking force and sticking quantity, it was discovered that traditional high-frequency electrosurgical scalpels encounter substantial sticking issues during surgical procedures. However, this problem was significantly alleviated with the use of an electrosurgical scalpel featuring a micro-nano textured functionalized surface, which was fabricated using nanosecond laser technology. To further corroborate this improvement, a comparative study was conducted using both a standard high-frequency electrosurgical scalpel and anti-sticking scalpels with microcolumn angles of 90°, 85°, 75°, and 60°. These scalpels were employed to perform cyclic cutting experiments on fresh pork liver, with 1, 10, and 20 cycles, under identical environmental conditions. The sticking quantity, sticking force, and the size of thermal damage wounds left on the fresh pork liver were then carefully compared and observed (Figure 9).

The experimental findings reveal that the micro-nano textured functionalized surface fabricated using nanosecond laser technology demonstrates a marked advantage in reducing sticking force and sticking quantity, with this benefit being particularly pronounced after multiple cutting cycles. For example, the lifespan of an electrosurgical scalpel equipped with anti-sticking characteristics is extended by over three-fold compared to a conventional high-frequency electrosurgical scalpel, while the frequency of cleaning needed during its usage is significantly reduced. Moreover, the variation in the microcolumn angle also influences the anti-sticking performance, with a general trend indicating that the smaller the angle β, the more effective the anti-sticking.

**Figure 10:**
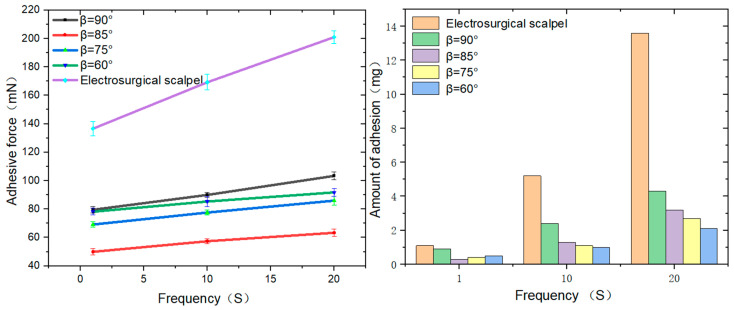
Comparison curves of adhesion amount versus adhesion force when the number of cycles are 1, 10, and 20.

Through adhesion force measurement experiments, we arrived at several pivotal findings: when performing a single electrosection on soft tissue, the functionalized surface of the anti-sticking electrosurgical scalpel with an inclined column angle of β at 85° demonstrated the most superior performance, achieving a minimum adhesion force of 50 mN. This represents a reduction of approximately 62.4%, compared to the high-frequency electrosurgical scalpel. In contrast, the surfaces with less effective anti-sticking properties had inclined column angles of β at 90° and 60°, yet still exhibited a decrease of around 45.6%, compared to the high-frequency variant. Upon the 20th electrosection of the biological tissue, the 85° inclined column angle continued to outperform, achieving a reduction over 90% versus the high-frequency electrosurgical scalpel. The 90° inclined column angle remained less effective, showing only a 52.4% reduction in adhesion force compared to the high-frequency scalpel. These data suggest an exponential growth trend in the differential adhesion force between the anti-sticking electrosurgical scalpel and the high-frequency electrosurgical scalpel. It is anticipated that after 40 cycles, the adhesion force of the anti-sticking electrosurgical scalpel will be diminished by 130%, providing robust evidence that the functionalized surface, prepared with nanosecond laser technology, possesses pronounced anti-sticking characteristics. Further examination of the thermal damage area at the incision site of fresh pork liver under different electrosurgical cutting cycles (S) revealed a positive correlation between the thermal damage area and the inclined column angle β. Specifically, the minimum thermal damage area is achieved when the inclined column angle β is 75°. Additionally, it was observed that the sticking amount increases as the inclined column angle β decreases. Compared to the traditional high-frequency electrosurgical scalpel, the sticking amount decreases by 316%, 425%, 503%, and 648%, respectively, for β values of 90°, 85°, 75°, and 60° (Figure 10).

These experimental results clearly demonstrate that the micro-nano textured functionalized surface prepared using nanosecond laser technology can significantly reduce sticking phenomena during surgical procedures with electrosurgical scalpels. It also diminishes the thermal damage caused by high-frequency currents. These findings confirm that the composite texture of microgrooves and microcolumns processed on the surface of electrosurgical scalpel blades using nanosecond laser technology, along with surface modification and the addition of lubricants, yields a functionalized surface with excellent anti-sticking properties and a small area of thermal damage to biological soft tissues.

Through cutting cycle tests, it was observed that the sticking force of the surface tissue significantly decreased, along with a substantial reduction in sticking quality. This result fully proves that the anti-sticking electrosurgical scalpel prepared using nanosecond laser technology possesses superior anti-sticking performance, maintaining stability over extended use and meeting the needs for sticking prevention in electrosurgical devices.

The excellent durability and efficient anti-sticking properties not only enhance surgical efficiency and safety but also reduce maintenance costs and surgical duration. This provides a more efficient and safe option for medical surgery.

## 3. Results and Discussion

By integrating the aforementioned research findings, we have leveraged nanosecond laser technology to design and fabricate a novel self-lubricating anti-sticking functionalized surface on the surface of a high-frequency electrosurgical scalpel. The achievements are as follows:

(1) This surface is composed of an array of microgrooves, each containing five rows of microcolumns, forming a unique micro-nano structure. On this micro-nano structure, we successfully grafted an OTS self-assembled coating, which plays a pivotal role in the effective diffusion, stable adhesion, and long-term retention of dimethyl silicone oil on the functionalized surface. The choice of dimethyl silicone oil as an efficient lubricant is due to its strong coating and stability, which are crucial for ensuring that the scalpel maintains good anti-sticking performance over extended use.
(2) The experiments also studied the unidirectional flow and reverse gravity spreading effects of three different liquids on the functionalized surface. The experimental results revealed that the functionalized surface not only possesses self-lubrication properties but can also achieve self-lubrication at a smaller angle with the horizontal plane. These experiments strongly confirm the feasibility of the lubricant in the prepared micro-nano texture for self-lubrication and self-supplementation.
(3) We measured the adhesion force and adhesion quantity of the anti-sticking electrosurgical scalpel compared to the high-frequency electrosurgical scalpel. The experiments showed that the adhesion force of the anti-sticking electrosurgical scalpel with the functionalized surface decreased up to 90% compared to the high-frequency electrosurgical scalpel, and the adhesion quantity reduced by 300–650%. These experimental results consistently demonstrate that the high-frequency electrosurgical scalpel with a self-lubricating anti-sticking surface prepared using nanosecond laser technology exhibits excellent anti-sticking performance.

These research findings not only provide new insights and methods for the design and fabrication of electrosurgical scalpels but also contribute significantly to enhancing the performance and safety of scalpels in practical applications. Looking forward, we anticipate that these discoveries will further advance the field of medical devices and bring more innovation and improvements to surgical procedures.

## Data Availability

The original contributions presented in the study are included in the article, further inquiries can be directed to the corresponding author.
